# Antibacterial efficacy of ethyl acetate fraction of *Psidium guajava* leaf aqueous extract on experimental *Escherichia coli* (O78) infection in chickens

**DOI:** 10.14202/vetworld.2015.358-362

**Published:** 2015-03-18

**Authors:** Y. A. Geidam, A. G. Ambali, P. A. Onyeyili, M. B. Tijjani, H. I. Gambo, I. A. Gulani

**Affiliations:** 1 Department of Veterinary Medicine, Faculty of Veterinary Medicine, University of Maiduguri, P.M.B. 1069, Maiduguri, Borno State, Nigeria; 2 Department of Veterinary Medicine, Faculty of Veterinary Medicine, University of Ilorin, Kwara State, Nigeria; 3 Department of Veterinary Physiology, Pharmacology and Biochemistry, College of Veterinary Medicine, Federal University of Agriculture, Makurdi, Benue State, Nigeria; 4 Department of Veterinary Physiology, Pharmacology and Biochemistry, Faculty of Veterinary Medicine, University of Maiduguri, P.M.B. 1069, Maiduguri, Borno State, Nigeria; 5 Department of Veterinary Pathology, Faculty of Veterinary Medicine, University of Maiduguri, P.M.B. 1069, Maiduguri, Borno State, Nigeria

**Keywords:** antibacteria, chickens *Escherichia coli*, efficacy, *Psidium guajava*

## Abstract

**Aim:**

This study was desingned to examine the efficacy of ethyl acetate fraction of aqueous extracted *Psidium guajava* leaves on chicks experimentally-infected with diarrheagenic strain of *Escherichia coli* O78.

**Methods:**

A total of 60 ISA brown male chicks were randomly divided into 6 Groups of ten chicks each in separate cages. Group A was not infected and not treated. Groups B, C and D were infected and treated with extracts at a dose of 25, 50 and 100 mg/kg respectively for 10 days. Group E was infected and treated with oxytetracycline while Group F was infected, but left untreated. Chicks from all groups were closely monitored for clinical signs, body weight change and fecal bacterial shedding load during the course of the experiment.

**Results:**

Diarrhea, vents pasted with feces, drop in feed intake accompanied by slow weight gain and decreased activity was observed in infected untreated groups. Groups treated with graded doses of the extract experienced a dose-dependent decreased in severity of the clinical signs shown compared to the infected untreated group. Bacterial shedding load was found to be lower in groups treated with the extract and oxytetracycline than those without intervention.

**Conclusion:**

Ethyl acetate soluble fraction of leaf extract of *Psidium guajava* effectively controlled diarrhea and decreased the severity of other clinical signs caused by experimental *E. coli* infections in chicks.

## Introduction

Colibacillosis refers to any localized or systemic infection caused entirely or partly by avian pathogenic *Escherichia coli* [1] and is one of the primary cause of morbidity, mortality and condemnation of carcasses in the poultry industry worldwide responsible for significant economic losses in poultry industry [2]. The major clinical signs complicating the virulence of the disease is profuse watery diarrhea and severe dehydration especially in diarrheagenic strains and prior to death, the affected birds become anorexic and emaciated with a case fatality rate of 12.67% [3].

Avian pathogenic *E. coli* strains are often resistant to approved antimicrobials for poultry including tetracyclines, sulfonamides [4], aminoglycosides [2] and ß-lactam antibiotics [5]. The emergence of resistant bacteria has created a major concern and an urgent need for new antibacterial agents [6].

Phytochemicals in herbs used in ethnomedicine are often exploited to discover novel lead compounds that is hoped to overcome the resistance produced by the pathogens, since they are in natural form or in pooled form of more than one molecule in the protoplasm of the plant [7]. Flavonoid content of *Psidium guajava* leaves has been observed to have antibacterial activities [8]. In Nigeria, the leaf extract has been used by herbalists to manage diarrhea, dysentery and as a wounds healing agent [9]. The *in-vitro* ethyl acetate soluble fraction of aqueous leaf extract of *P. guajava* at a low concentration of 12.5 mg/ml was found to inhibit some Gram-positive and Gram-negative bacterial organisms [10].

Existing antibiotics are faced with the challenge of bacterial resistance and hence the need for research into finding new novel antibiotics. Preliminary clinical trial on experimental animals is the first stage for determining claim of efficacy of a new promising drug. Our previous finding [10] showed that the extract demonstrated *in vitro* antibacterial activity, and this informed the research to determine the efficacy of *P. guajava* in experimentally infected chicks.

## Materials and Methods

### Ethical approval

This research was approved by the research animal use and care Committee, University of Maiduguri.

### Extract preparation

Fresh leaves of *P. guajava* was collected, air-dried and ground into a fine powder. The powdered sample was then soaked in distilled water and extracted using reflux method. The filtrate obtained was concentrated *in vacou* and yielded a brown colored solid, aqueous extract weighing 33.75% of its original sample weight. The resulting aqueous extract was further fractioned exhaustively with ethyl acetate solvent as described previously [11,12]. The ethyl acetate fraction of the aqueous extract was used as a test sample to investigate *in vivo* antibacterial efficacy, and oxytetracycline (Terramycin^®^, Pfizer, Inc. USA) was the standard antibiotics.

### Experimental birds, groupings and procedures

Sixty (60) ISA brown male chicks were used for the study. They were kept in the Animal House of Department of Veterinary Medicine, University of Maiduguri and fed with chick mash (Vital Feed Ltd., Jos, Nigeria) and water ad libitum. The chicks were allowed to acclimatize in their new environment for 10 days before the commencement of the experiment. They were randomly divided into 6 Groups (Groups A, B, C, D, E and F) of 10 chicks each and were kept in separate cages. Group A served as the control group (no infection and no treatment). Groups B, C, and D were infected with *E. coli* and were treated with the extract at doses of 25, 50 and 100 mg/kg respectively. Group E was infected but treated with oxytetracycline (standard drug) at 1 g/l of drinking water, whereas Group F was infected but left untreated.

Avian pathogenic *E. coli* strain O78 isolated from sick rural chickens with profuse watery diarrhea was used to infect the birds. The infective inoculum was prepared from an overnight nutrient broth culture of the isolate and the number of organisms estimated as colony forming units (CFU) per ml of suspension in sterile nutrient broth matched with a standard MacFarland Scale. Colony count was performed on the diluted culture to achieve a titer of 10^8^ CFU/ml [13]. All chicks were starved overnight before the experimental infection. The chicks were each inoculated orally with 1 ml of the inoculum. The uninfected control birds were sham-inoculated with sterile broth.

Cloacal swabs were collected from the infected and control chicks before the infection and on days 2,4,6,8 and 10 post-infection (PI) and transferred into normal saline. Serial dilution was made from 1:10 to 1:10^9^ and then 0.1 ml of 1:10^9^ was inoculated onto eosin methylene blue and MacConkey agar for isolation, identification and quantification of *E. coli* shedding (shedding load) per chick. Slide agglutination test on randomly selected colonies were performed with specific anti-serum against the O78 strain.

Chicks from all groups were closely monitored for clinical signs of infection. Treatment with the extract as well as with the standard drug started simultaneously in all the groups as soon as clinical signs of infection were obvious in the infected groups. The number of wet droppings on the floor, quantity of feeds consumed, level of activity and body weights of chicks in each groups were recorded before the infection and at days 2,4,6,8, and 10 PI. Two chicks each from all the groups were sacrificed after 7 days of treatment and sections of small intestines was obtained and fixed in 10% formalin thereafter, embedded in paraffin wax and sections cut at 5 µm thickness and stained with hematoxylin and eosin. The slides obtained were examined for histopathological lesions.

### Statistical analysis

Graphpad In Stat [14] statistical package was used to compare data for statistical significance using Analysis of Variance. The mean viable count of *E. coli* and mean body weights between groups was compared. p<0.05 was considered as significant. Dunnett multiple comparison post-test was used for analysis of the mean values.

## Results

### Clinical signs

**Figure-1:**
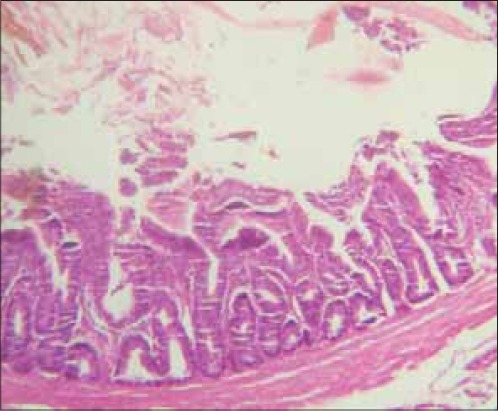
Photomicrograph of small intestine of chick from infected untreated group at 7 days post-infection showing severe villous collapse (stunting, matting and fusion of villi) (H and E; × 350).

**Figure-2:**
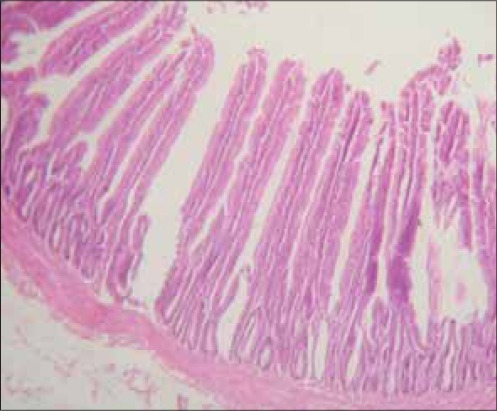
Photomicrograph of small intestine of infected treated chick with 100 mg/kg of ethyl acetate fraction of *Psidium guajava* treated group at 7 days of treatment showing evidence of recovery to normalcy of the intestinal villi (H and E; × 350)

**Figure-3:**
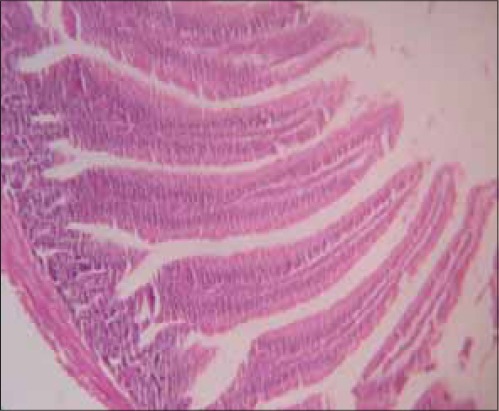
Photomicrograph of small intestine of chick from negative control group (uninfected untreated chick) (H and E; ×350). Note the normal architecture of the intestine

There was a remarkable improvement in severity of diarrhea in birds that were given the extract compared to infected untreated control group. The intestine of chicks sacrificed from the infected untreated group showed severe villous collapse with stunting, matting and fusion of villi (Figure-1), but there was remarkable improvement in those lesions observed in (100 mg/kg) extract treated chicks (Figure-2) while Figure-3 showed the histological section of a normal intestine. Chicks from infected untreated groups showed sign of depression as suggested by decrease in activity and in appetence as demonstrated by a drop in feed intake. The pattern of feed intake showed that level of inappetence had drastically reduced to near normal value as the treatment with the graded doses of the extract continued through day 6. Decreased activity in the early experiment with subsequent improvement after the treatment with the extract demonstrated the efficacy against depression induced by the causative agent (Table-1).

**Table-1:** Major clinical signs of chicks infected with *E. coli* O78 and treated with graded doses of ethyl acetate fraction of *Psidium guajava* leaf extract.

Clinical parameter	Treatment Group	Alternate day observations during treatment period (day)				

		2^nd^	4^th^	6^th^	8^th^	10^th^
Diarrhoea (number of wet droppings)	Negative control	2	4	4	6	6
	25 mg/kg	10	12	10	10	8
	50 mg/kg	12	10	8	7	6
	100 mg/kg	9	10	7	7	6
	Oxytetracycline	12	9	6	6	5
	Infected untreated	11	10	11	12	10
Feed Consumption (g)	Negative control	30	34	40	43	48
	25 mg/kg	32	26	32	37	41
	50 mg/kg	29	24	34	38	43
	100 mg/kg	27	25	37	43	45
	Oxytetracycline	31	28	38	44	47
	Infected untreated	30	21	22	20	18
Level of Activity	Negative control	+ + +	+ + +	+ + +	+ + +	+ + +
	25 mg/kg	+	+	+ +	+ +	+ + +
	50 mg/kg	+	+ +	+ +	+ + +	+ + +
	100 mg/kg	+	+ +	+ +	+ + +	+ + +
	Oxytetracycline	+	+ +	+ + +	+ + +	+ + +
	Infected untreated	+	+	+	+	+

+=Little activity, ++=Moderate activity, +++=Normal activity

### Effects of treatment with graded doses of ethyl acetate fraction of the extract on body weight of chicks infected with *E. coli* O78.

Table-2 shows the result of the various treatments on the body weight of the birds. *E. coli* infection affected body weight gain of infected birds. There was marked improvement in body weight at day 8 in the negative control group from 60±8 g to 85±7 g but the extract at 50 and 100 mg/kg, and oxytetracycline group induced much earlier weight gain at day 6 from initials 49±8, 50±9 and 50±6 g to 65±8 g for both the extract groups and 71±8 g for oxytetracycline group. This pattern was maintained throughout the experiment.

**Table-2:** Mean body weight of chicks infected with *E. coli* and treated with graded doses of ethyl acetate fraction of *Psidium guajava* leaf extract.

Groups (mg/kg)	Body weight (X±SD) (g) days of treatment					

	0	2	4	6	8	10
Negative control	60±8	65±6	68±6	74±8	85±7^s^	101±8^s^
25	58±9	65±8	65±3	70±6	80±9^s^	90±5^s^
50	49±8	54±7	58±6	65±8^s^	69±7^s^	75±10^s^
100	50±9	53±8	61±9	65±8^s^	69±8^s^	72±11^s^
Oxytetracycline	50±6	55±7	62±7^s^	67±6^s^	71±8^s^	78±6^s^
Infected untreated	53±8	59±7	61±7	61±5	63±9	63±8

Values in a row with superscript s are significantly higher than day 0 value

### Effects of treatment with graded doses of ethyl acetate fraction of the extract on fecal bacterial shedding load of chicks infected with *E. coli* O78

Results of bacterial shedding load, shown in Table-3, indicated that in 25 mg/kg treated group, the initial shedding load before the beginning of the experiment (day 2) was 65±4 and was maintained at statistically non-significant level of 64±3, 59±5, 55±3 and 58±4 at 4^th^, 6^th^, 8^th^ and 10^th^ days, respectively. At higher doses of 50 mg/kg and 100 mg/kg, the pre-treatment bacterial shedding load value of 65±4 and 68±4 CFU/ml did not significantly change at day 4 and 6, but was significantly reduced to 46±3 and 45±6 CFU/ml at the 8^th^ day and then 44±3 and 43±4 CFU/ml at the 10^th^ day, respectively. The group treated with the standard antibiotics showed a similar pattern when compared to the group treated with 100 mg/kg of the extract.

**Table-3:** Mean bacterial shedding load of chicks infected with *E. coli* and treated with various doses of ethyl acetate fraction of *Psidium guajava* leaf extract.

Groups (mg/kg)	Bacterial shedding load (×10^9^) (X±SD) (CFU/ml) Days of treatment					

	0	2	4	6	8	10
Negative control	60±5	61±3	61±6	62±4	63±3	63±2
25	60±4	65±4	64±3	59±5	55±3	58±4
50	60±5	65±4	61±6	58±4	46±3^s^	44±3^s^
100	60±4	68±4	62±3	57±4	45±6^s^	43±4^s^
Oxytetracycline	60±5	65±6	58±5	50±6	44±4^s^	42±6^s^
Infected untreated	60±4	71±6^a^	76±5^b^	86±5^b^	88±6^b^	86±4^b^

S=Values in the same row are significantly lower than day zero, b=Values in the same row are significantly higher than day 0

## Discussion

Clinical recovery of *E. coli* infected chicks treated with ethyl acetate extracts at all the graded doses used was evidenced by decrease in the severity of the disease signs, which were supported by histological and microbiological evidence with improvement of weight gain clearly demonstrated the efficacy of ethyl acetate fraction of *P. guajava* aqueous extract. Some limiting factors that inhibit a compound with *in vitro* activity to show similar biological activity following oral administration are due to absorption failure or inactivation as a result of enzymatic degradation, change in pH or temperature in the gastro intestinal tract [15,16].

Ethyl acetate fraction of *P. guajava* was able to replicate *in-vitro* findings of antibacterial activity and this signifies that the extract is stable in the gastrointestinal tract and since the pathogenicity of diarrheagenic strains of *E. coli* O78 is mainly in the intestine, pharmacokinetic factor may not influence the activity of the extract and this could be responsible for the extract’s observed efficacy.

Phytochemical constituent of the extract was reported [10] to have tannins and they are reputed to have anti-diarrheic properties due to their ability to precipitate protein and consequently give feces a firmer consistency [17]. Similar finding was reported [18] on Detarium microcarpum stem aqueous extract where the plant was found to possess tannins and effectively stopped experimentally-induced diarrhea in albino rats.

Some antibiotics with mechanism of action of inhibiting bacterial protein syntheses such as erythromycin and chloramphenicol have the characteristic bacteriostatic activity at lower doses and bactericidal activity at higher doses [19,20]. The observation that the extract did not change significantly from the baseline value signify that it stabilized bacterial shedding load at low doses, and the significant reduction from the baseline value in bacterial shedding load at higher doses; could suggest that extract exhibits both bacteriostatic and bactericidal activities at lower and higher doses respectively.

Based on the findings of this research showing clinical recovery with corresponding findings in tissue histology and microbiological evidence suggests that the extract is effective in the management of diarrhea caused by *E. coli*. Further research needs to be carried out to find out its mechanism of action and its utility in clinical practice.

## Conclusion

It is concluded that the ethyl acetate soluble fraction of *P. guajava* leaf effectively controlled diarrhea and decreased the severity of other clinical signs and bacterial load caused by experimental *E. coli* infections in chicks. Further research is needed to ascertain the mechanism of action with the view of its application in clinical practice.

## Authors’ Contributions

YAG, AGA and PAO conceptualized and designed the study. AGA and POA were involved in the experimental infection and fractional distillation of the leaf extract, respectively. YAG was involved in all aspect of the research. MBT conducted the phytochemical analysis of the extract. HIG coordinated the research and interpreted the histopathological findings. IAG has done microbiological analysis. All authors participated in draft and revision of the manuscript. All authors read and approved the final manuscript.
